# Multiview Layer Fusion Model for Action Recognition Using RGBD Images

**DOI:** 10.1155/2018/9032945

**Published:** 2018-06-20

**Authors:** Pongsagorn Chalearnnetkul, Nikom Suvonvorn

**Affiliations:** Department of Computer Engineering, Faculty of Engineering, Prince of Songkla University, Hat Yai, Songkhla 90110, Thailand

## Abstract

Vision-based action recognition encounters different challenges in practice, including recognition of the subject from any viewpoint, processing of data in real time, and offering privacy in a real-world setting. Even recognizing profile-based human actions, a subset of vision-based action recognition, is a considerable challenge in computer vision which forms the basis for an understanding of complex actions, activities, and behaviors, especially in healthcare applications and video surveillance systems. Accordingly, we introduce a novel method to construct a* layer feature model* for a profile-based solution that allows the fusion of features for multiview depth images. This model enables recognition from several viewpoints with low complexity at a real-time running speed of 63 fps for four profile-based actions: standing/walking, sitting, stooping, and lying. The experiment using the Northwestern-UCLA 3D dataset resulted in an average precision of 86.40%. With the i3DPost dataset, the experiment achieved an average precision of 93.00%. With the PSU multiview profile-based action dataset, a new dataset for multiple viewpoints which provides profile-based action RGBD images built by our group, we achieved an average precision of 99.31%.

## 1. Introduction

Since 2010, action recognition methods have been increasingly developed and have been gradually introduced in healthcare applications, especially for monitoring the elderly. Action analysis plays an important role in the investigation of normal or abnormal events in daily-life activities. In such applications, privacy and convenience of usage of chosen technologies are two key factors that must be thoroughly considered. The pattern of recognized actions is an important function of a system for monitoring complex activities and behaviors which consist of several brief actions constituting a longer-term activity outcome. For example, a sleeping process involves standing/walking, sitting, and lying actions; and a falling process includes all actions mentioned above except sitting.

Recently, two main approaches have been studied and proposed for determining these actions: a wearable sensor-based technique and a vision-based technique.

Wearable inertial sensor-based devices have been used extensively in action recognition due to their small size, low power consumption, low cost, and the ease with which they can be embedded into other portable devices, such as mobile phones and smart watches. An inertial sensor used for performing navigation commonly comprises motion and rotation sensors, (e.g. accelerometers and gyroscopes). It provides the path of movement, viewpoint, velocity, and acceleration of the tracked subject. Some research studies have used wearable sensors [1–3], mobile phones [4–7], and smart watches [8] for recognizing different actions. In some research, the focus was on detection of abnormal actions, such as falling [9–11], or on reporting status for both normal and abnormal situations [12]. To recognize complex actions, moreover, several sensors must be embedded at different positions on the body. The only limitation of inertial sensors is the inconvenience presented because sensors must eventually be attached to the body, which is uncomfortable and cumbersome.

For vision-based techniques, many studies emphasize using either a single-view or multiview approach for recognizing human actions.

In a single-view approach, four types of feature representation have been used: (1) joint-based/skeleton-based, (2) motion/flow-based, (3) space-time volume-based, and (4) grid-based:

1. Joint-based/skeleton-based representation defines the characteristics of human physical structure and distinguishes its actions, for example, multilevel of joints and parts from posing features [13], the Fisher vector using skeletal quads [14], spatial-temporal feature of joints-mHOG [15], Lie vector space from a 3D skeleton [16], invariant trajectory tracking using fifteen joints [17], histogram bag-of-skeleton-codewords [18], masked joint trajectories using 3D skeletons [19], posture features from 3D skeleton joints with SVM [20], and star skeletons using HMMs for missing observations [21]. These representations result in clear human modeling, although the complexity of joint/skeleton estimation requires good accuracy from tracking and prediction.
2. Motion/flow-based representation is a global feature-based method using the motion or flow of an object, such as invariant motion history volume [22], local descriptors from optical-flow trajectories [23], KLT motion-based snippet trajectories [24], Divergence-Curl-Shear descriptors [25], hybrid features using contours and optical flow [26], motion history and optical-flow images [27], multilevel motion sets [28], projection of accumulated motion energy [29], pyramid of spatial-temporal motion descriptors [30], and motion and optical flow with Markov random fields for occlusion estimation [31]. These methods do not require accurate background subtractions but make use of acquired, inconstant features that need strategy and descriptors to manage.
3. Volume-based representations are modeled by stacks of silhouettes, shapes, or surfaces that use several frames to build a model, such as space-time silhouettes from shape history volume [32], geometric properties from continuous volume [33], spatial-temporal shapes from 3D point clouds [34], spatial-temporal features of shapelets from 3D binary cube space-time [35], affine invariants with SVM [36], spatial-temporal micro volume using binary silhouettes [37], integral volume of visual-hull and motion history volume [38], and saliency volume from luminance, color, and orientation components [39]. These methods acquire a detailed model but must deal with high dimensions of features which require accurate human segmentation without the background.
4. Grid-based representations divide the observation region of interest into cells, a grid, or overlapped blocks to encode local features, for example, a grid or histogram of oriented rectangles [40], flow descriptors from spatial-temporal small cells [41], histogram of local binary patterns from a spatial grid [42] and rectangular optical-flow grid [43], codeword features for histograms of oriented gradients and histograms of optical flow [44], 3D interest points within multisize windows [45], histogram of motion gradients [46], and combination of motion history, local binary pattern, and histogram of oriented gradients [47]. This method is simple for feature modeling in the spatial domain, but it must deal with some duplicate and insignificant features.

Although the four types of representation described in the single-view approach are generally good, in monitoring a large area, one single camera will lose its ability to determine continuous human daily-life actions due to view variance, occlusion, obstruction, and lost information, among others. Thus, a multiview approach is introduced to lessen the limitations of a single-view approach.

In the multiview approach, methods can be categorized into 2D and 3D methods.

Examples of the 2D methods are layer-based circular representation of human model structure [48], bag-of-visual-words using spatial-temporal interest points for human modeling and classification [49], view-invariant action masks and movement representation [50], R-transform features [51], silhouette feature space with PCA [52], low-level characteristics of human features [53], combination of optical-flow histograms and bag-of-interest-point-words using transition HMMs [54], contour-based and uniform local binary pattern with SVM [55], multifeatures with key poses learning [56], dimension-reduced silhouette contours [57], action map using linear discriminant analysis on multiview action images [58], posture prototype map using self-organizing map with voting function and Bayesian framework [59], multiview action learning using convolutional neural networks with long short term memory [60], and multiview action recognition with an autoencoder neural network for learning view-invariant features [61].

Examples of the 3D method, where the human model is reconstructed or modeled from features between views, are pyramid bag-of-spatial-temporal-descriptors and part-based features with induced multitask learning [62], spatial-temporal logical graphs with descriptor parts [63], temporal shape similarity in 3D video [64], circular FFT features from convex shapes [65], bag-of-multiple-temporal-self-similar-features [66], circular shift invariance of DFT from movement [67], and 3D full body/pose dictionary features with convolutional neural networks [68]. All of these 3D approaches attempt to construct a temporal-spatial data model that is able to increase the model precision and, consequently, raise the accuracy of the recognition rate.

The multiview approach, however, has some drawbacks. The methods need more cameras and hence are more costly. It is a more complex approach in terms of installation, camera calibration between viewpoints, and model building and hence is more time-consuming. In actual application, however, installation and setup should be simple, flexible, and as easy as possible. Systems that are calibration-free or automatically self-calibrating between viewpoints are sought.

One problem facing a person within camera view, be it one single camera or a multitude of cameras, is that of privacy and lighting conditions. Vision-based and profile-based techniques involve the use of either RGB or non-RGB. The former poses a serious problem to privacy. Monitoring actions in private areas using RGB cameras make those under surveillance feel uncomfortable because the images expose more clearly their physical outlines. As for lighting conditions, RGB is also susceptible to intensity; images often deteriorate in dim environments. The* depth approach* helps solve both problems; a coarse depth profile of the subject is adequate for determining actions, and depth information can prevent illumination change issues, which are a serious problem in real-life applications of round-the-clock surveillance. The depth approach that is adopted in our research together with a multiview arrangement is considered worthy of more costly installation than the single-view approach.

A gap that needs attention for most multiview, non-RGB results is that of perspective robustness, or viewing-orientation stability, and model complexity. Under a calibration-free setup, our research aims to contribute to the development of a fusion technique that is robust and simple in evaluating the depth profile of human action recognition. We have developed a* layer fusion model* in order to fuse depth profile features from multiviews and to test our technique on a triple dataset of validation and efficiency. The three datasets tested are the Northwestern-UCLA dataset, the i3DPost dataset, and the PSU dataset for multiview action from various viewpoints.

The following sections detail our model, its results, and comparisons.

## 2. Layer Fusion Model

**Figure 1:**
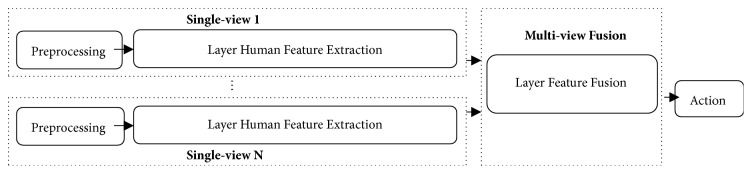
Overview of layer fusion model.

Our* layer fusion model* is described in three parts: (1) preprocessing for image quality improvement; (2) human modeling and feature extraction using a single-view* layer feature extraction module*; and (3) fusion of features from any view into one single model using* layer feature fusion module* and classifying to actions. The system overview is shown in Figure 1.

### 2.1. Preprocessing

**Figure 2:**
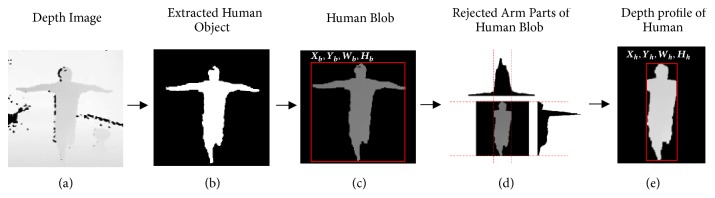
Preprocessing and human depth profile extraction; (a) 8-bit depth image acquired from depth camera; (b) motion detected output from mixture-based Gaussian model for background subtraction; (c) blob position: consists of top-left position (X_b_, Y_b_) and width (W_b_) and height (H_b_); (d) rejected arm parts of human blob; (e) depth profile and position of human blob: X_h_, Y_h_, W_h_, and H_h_.

The objective of preprocessing is to segregate human structure from the background and to eliminate arm parts before extracting the features, as depicted in Figure 2. In our experiment, the structure of the human in the foreground is extracted from the background by applying motion detection using mixture of Gaussian model segmentation algorithms [69]. The extracted motion image (***I***_***m***_) (Figure 2(b)), from the depth image (***I***_***d***_) (Figure 2(a)), is assumed to be the human object. However,***I***_***m***_ still contains noise from motion detection, which has to be reduced by morphological noise removal operator. The depth of the extracted human object, defined by its depth values inside the object, is then improved (***I***_***mo***_) as determined by intersecting*** I***_***d***_ and*** I***_***m***_ using the AND operation:*** I***_***mo***_ =*** I***_***m***_&***I***_***d***_.

The human blob, with bounding rectangle coordinates* X*_*h*_ and* Y*_*h*_, width* W*_*h*_, and height* H*_*h*_ as shown in Figure 2(c), is located using a contour approximation technique. However, our action technique emphasizes only the profile structure of the figure, while hands and arms are excluded, as seen in Figure 2(d), and the obtained structure is defined as the figure depth profile (***I***_***mo***_) (Figure 2(e)) in further recognition steps.

### 2.2. Layer Human Feature Extraction

**Figure 3:**
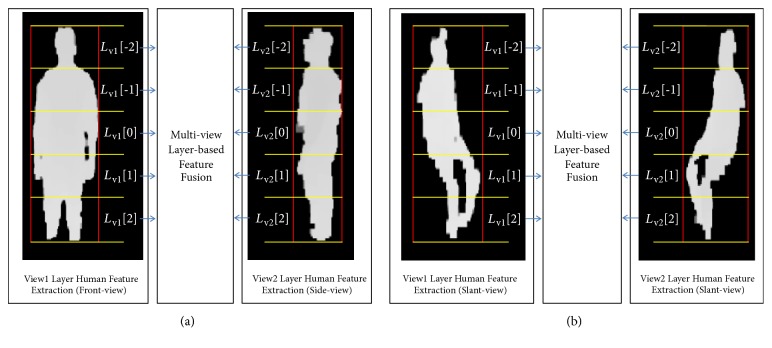
Layered human model for multiview fusion: (a) sampled layer model for standing; (b) sampled layer model for sitting.

We model the depth profile of a human in a layered manner that allows extraction of specific features depending on the height level of the human structure, which possesses different physical characteristics. The depth profile is divided vertically into odd-numbered layers (e.g., 5 layers, as shown in Figure 3) with a specific size, regardless of the distance, perspective, and views, which would allow features of the same layer from all views to be fused into reliable features.

The human object in the bounding rectangle is divided into equal layers to represent features at different levels of the structure,* L*[*k*]* | k ∈*{-*N, -N+*1*, -N+*2*,...,*0, 1, 2*, N-*2*, N-*1, *N*}. The total number of layers is 2*N+*1, where* N* is the maximum number of upper or lower layers. For example, in Figure 3, the human structure is divided into five layers (*N* equals 2): two upper, two lower, and one center; thus the layers consist of {*L*[*-*2],* L*[-1],* L*[0],* L*[+1], and* L*[+2]}. The horizontal boundaries of all layers, the red vertical lines, are defined by the left and right boundaries of the human object. The vertical boundaries of each layer, shown as yellow horizontal lines, are defined by the top *y*_*T*_[*k*] and the bottom *y*_*B*_[*k*] values that can be computed as follows:

(1) yTk=Hhk+N2N+1+1

(2) yBk=Hhk+N+12N+1

The region of interest in layer *k* is defined as *x* = 0 to W_h_ and *y* = *y*_*T*_[*k*] to *y*_*B*_[*k*].

According to the model, features from the depth profile human object can be computed along with layers as concatenated features of every segment using basic and statistical properties (e.g., axis, density, depth, width, and area). Depth can also be distinguished for specific characteristic of actions.

In our model, we define two main features for each layer, including the* density* (*ρ*[*k*]) and* weighted depth density* (Z[*k*]) of layers. In addition, the* proportion value* (*P*_v_) is defined as a global feature to handle the horizontal action.

#### 2.2.1. Density of Layer

The* density of layer* (*ρ*[*k*]) indicates the amount of object at a specific layer, which varies distinctively according to actions, and can be computed as the number of white pixels in the layer, as shown in the following equation:

(3) $$\begin{array}{c} \rho^{\prime }\left[\;k\;\right]=\overset{y_{B}\left[k\right]}{\underset{y=y_{T}\left[k\right]}{\sum }}\;\overset{W_{h}}{\underset{x=0}{\sum }}I_{m}\left(\;x,y\;\right) \end{array}$$

In multiple views, different distances between the object and the cameras would affect *ρ*′[*k*]. Objects close to a camera certainly appear larger than when further away, and thus *ρ*′[*k*] must be normalized in order for these to be fused. We use the maximum value of the perceived object and normalize it, employing the following equation:

(4) $$\begin{array}{c} \rho \left[\;k\;\right]=\frac{\rho^{\prime }\left[k\right]}{arg\;max\left(\rho^{\prime }\left[k\right]\right)} \end{array}$$

#### 2.2.2. Weighted Depth Density of Layer

An inverse depth density is additionally introduced to improve the pattern of density feature. The procedure is comprised of two parts: inverse depth extraction and weighting for density of the layer.

At the outset, depth extraction is applied to the layer profile. The depth profile reveals the surface of the object that indicates rough structure ranging from 0 to 255 or from near to far distances from the camera. According to perspective projection varying in a polynomial form, a depth value at a near distance, for example, from 4 to 5, has a much smaller real distance than a depth value at a far distance, for example, from 250 to 251. The real-range depth of the layer (D′[*k*]) translates the property of 2D depth values to real 3D depth values in centimeters. The real-range depth better distinguishes the depth between layers of the object—different parts of the human body—and increases the ability to classify actions. A polynomial regression [70] has been used to convert the depth value to real depth, as described in the following equation:

(5) fc′x=1.2512e−10x6−1.0370e−7x5+3.5014e−5x4−0.0061x3+0.5775x2−27.6342x+5.6759e2

In addition, the (D′[*k*]) value of each layer is represented by the converted value of every depth value averaged in that layer, as defined in the following equation:

(6) $$\begin{array}{c} D^{\prime }\left[\;k\;\right]=f_{c}^{\prime }\left(\;\frac{\sum_{y=y_{T}\left[k\right]}^{y_{B}\left[k\right]}\sum_{x=0}^{W_{h}}I_{mo}\left(x,y\right)}{\rho \left[k\right]}\;\right) \end{array}$$

Numerically, to be able to compare the depth profile of human structure from any point of view, the D′[*k*] value needs to be normalized using its maximum value over all layers by employing the following equation:

(7) $$\begin{array}{c} D\left[\;k\;\right]=\frac{D^{\prime }\left[k\right]}{arg\;max\left(D^{\prime }\left[k\right]\right)} \end{array}$$

In the next step, we apply the inverse real-range depth (D_*i*_[*k*]), hereafter referred to as the inverse depth, for weighting the density of the layer in order to enhance the feature *ρ*[*k*] that increases the probability of classification for certain actions, such as sitting and stooping. We establish the inverse depth, as described in (8), to measure the hidden volume of body structure which distinguishes particular actions from others: for example, in Table 1, in viewing stooping from the front, the upper body is hidden but the value of D_*i*_[*k*] can reveal the volume of this zone; and in viewing sitting from the front, the depth of the thigh will reveal the hidden volume compared to other parts of the body.

(8) $$\begin{array}{c} D_{i}\left[\;k\;\right]=\left(\;\frac{D\left[k\right]-arg\;max\left(D\left[k\right]\right)}{arg\;min\left(D\left[k\right]\right)-arg\;max\left(D\left[k\right]\right)}\;\right) \end{array}$$

The inverse depth density of layers (Q[*k*]) in (9) is defined as the product of the inverse depth (D_*i*_[*k*]) and the density of the layer (*ρ*[*k*]).

(9) $$\begin{array}{c} Q\left[\;k\;\right]=\left(\;D_{i}\left[\;k\;\right]\;\right)\left(\;\rho \left[\;k\;\right]\;\right) \end{array}$$

We use learning rate *α* as an adjustable parameter that allows balance between Q[*k*] and *ρ*[*k*]. In this configuration, when the pattern of normalized inverse depth D_i_[k] is close to zero, Q[*k*] is close to *ρ*[*k*]. Equation (10) is the* weighted depth density of layers* (Z[*k*]), adjusted by the learning rate on Q[*k*] and *ρ*[*k*].

(10) $$\begin{array}{c} Ζ\left[\;k\;\right]=\left(\;1-\alpha \;\right)\left(\;Q\left[\;k\;\right]\;\right)+\left(\;\alpha \;\right)\left(\;\rho \left[\;k\;\right]\;\right) \end{array}$$

As can be deduced from Table 2, the weighted depth density of layers (Z[*k*]) improves the feature pattern for better differentiation for 13 out of 20 features, is the same for 5 features (I through V on standing-walking), and is worse for 2 features (VIII on side-view sitting and X on back-view sitting). Thus, Z[*k*] is generally very useful, though the similar and worse outcomes would require a further multiview fusion process to distinguish the pattern.

#### 2.2.3. Proportion Value

Proportion value (*P*_v_) is a penalty parameter of the model to indicate roughly the proportion of object vertical actions distinguished from horizontal actions. It is the ratio of the width *W*_*h*_ and the height *H*_*h*_ of the object in each view (see the following equation):

(11) $$\begin{array}{c} P_{v}=\frac{W_{h}}{H_{h}} \end{array}$$

Table 1 mentioned earlier shows the depth profile of action and its features in each view. In general, the feature patterns of each action are mostly similar, though they do exhibit some differences depending on the viewpoints. It should be noted here that the camera(s) are positioned at 2 m above the floor, pointing down at an angle of 30° from the horizontal line.

Four actions in Table 1, standing/walking, sitting, stooping, and lying, shall be elaborated here. For standing/walking, the features are invariant to viewpoints for both density (*ρ*[*k*]) and inverse depth (D_*i*_[*k*]). The D[*k*] values slope equally for every viewpoint due to the position of the camera(s). For sitting, the features vary for *ρ*[*k*] and D[*k*] according to their viewpoints. However, the patterns of sitting for front and slant views are rather similar to standing/walking. D[*k*] from some viewpoints indicates the hidden volume of the thigh. For stooping, the *ρ*[*k*] patterns are quite the same from most viewpoints, except for the front view and back view, due to occlusion of the upper body. However, D[*k*] reveals clearly the volume in stooping. For lying, the *ρ*[*k*] patterns vary depending on the viewpoints and cannot be distinguished using layer-based features. In this particular case, the proportion value (*P*_v_) is introduced to help identify the action.

### 2.3. Layer-Based Feature Fusion

In this section, we emphasize the fusion of features from various views. The pattern of features can vary or self-overlap with respect to the viewpoint. In a single view, this problem leads to similar and unclear features between actions. Accordingly, we have introduced a method for the fusion of features from multiviews to improve action recognition. From each viewpoint, three features of action are extracted: (1) density of layer (*ρ*[*k*]), (2) weighted depth density of layers (Z[*k*]), and (3) proportion value (*P*_v_). We have established two fused features as the combination of width, area, and volume of the body structure from every view with respect to layers. These two fused features are the mass of dimension (*ω*[*k*]) and the weighted mass of dimension ($\overset{-}{\omega }[k]$).

(a) The mass of dimension feature (*ω*[*k*]) is computed from the product of density of layers (*ρ*[*k*]) in every view, from *ν* = 1 (the first view) to *ν* = *d* (the last view), as shown in the following equation:
  (12) $$\begin{array}{c} \omega \left[\;k\;\right]=\overset{d}{\underset{v=1}{\prod }}\left(\;\rho_{\left(v=i\right)}\left[\;k\;\right]\;\right) \end{array}$$
(b) The weighted mass of dimension feature ($\overset{-}{\omega }[k]$) in (13) is defined as the product of the weighted depth density of layers (Z[*k*]) from every view.
  (13) $$\begin{array}{c} \overset{-}{\omega }\left[\;k\;\right]=\overset{d}{\underset{v=1}{\prod }}\left(\;{Ζ}_{\left(v=i\right)}\left[\;k\;\right]\;\right) \end{array}$$

In addition, the maximum proportion value is selected from all views (see (14)) which is used to make feature vector.

(14) $$\begin{array}{c} P_{\text{m}}=\text{argmax (}P_{\text{v}\mathrm{(v = 1)}},P_{\text{v}\mathrm{(v = 2),}}P_{\text{v}\mathrm{(v = 3),}}\text{…},P_{\text{v}\mathrm{(v = d)}}\text{)} \end{array}$$

Two feature vectors, a nondepth feature vector and a depth feature vector, are now defined for use in the classification process.

The nondepth feature vector is formed by concatenating the mass of dimension features (*ω*[*k*]) and the maximum proportion values as follows.

(15) ω−N,ω−N+1,ω−N+2,…,ω0,ω1,ω2,…,ωN,Pm

The depth feature vector is formed by the weighted mass of dimension features ($\overset{-}{\omega }[k]$) concatenated with the maximum proportion values (*P*_m_) as follows:

(16) ω−−N,ω−−N+1,ω−−N+2,…,ω−0,ω−1,ω−2,…,ω−N,Pm

Table 2 shows the weighted mass of dimension feature ($\overset{-}{\omega }[k]$) fused from the weighted depth density of layers (Z[*k*]) from two cameras. The fused patterns for standing-walking in each view are very similar. For sitting, generally the patterns are more or less similar except in the back view due to the lack of leg images. The classifier, however, can differentiate posture using the thigh part, though there are some variations in the upper body. For stooping, the fused patterns are consistent in all views: heap in the upper part but slightly different in varying degrees of curvature. For lying, all fused feature patterns are different. The depth profiles, particularly in front views, affect the feature adjustment. However, the classifier can still distinguish appearances, because generally patterns of features in the upper layers are shorter than those in the lower layers.

## 3. Experimental Results

Experiments to test the performance of our method were performed on three datasets: the PSU (Prince of Songkla University) dataset, the NW-UCLA (Northwestern-University of California at Los Angeles) dataset, and the i3DPost dataset. We use the PSU dataset to estimate the optimal parameters in our model, such as the number of layers and the adjustable parameter *α*. The tests are performed on single and multiviews, angles between cameras, and classification methods. Subsequently, our method is tested using the NW-UCLA dataset and the i3DPost dataset, which is set up from different viewpoints and angles between cameras to evaluate the robustness of our model.

### 3.1. Experiments on the PSU Dataset

**Figure 4:**
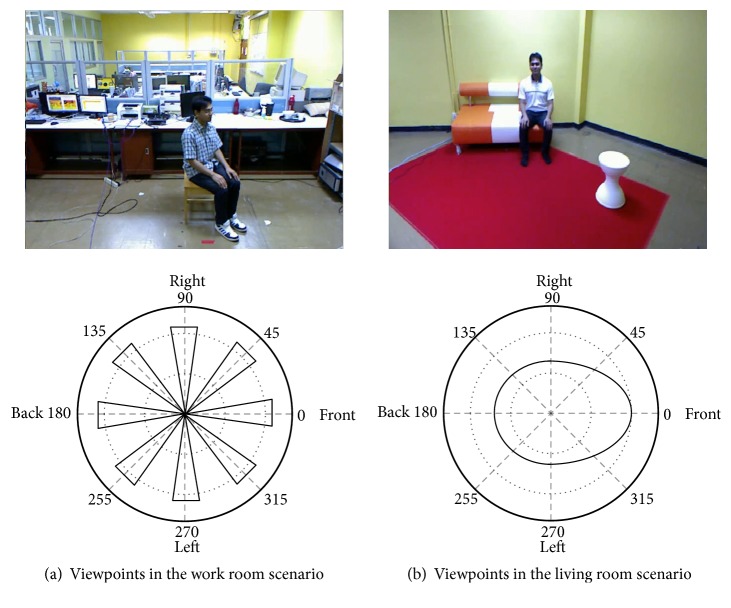
Example of two multiview scenarios of profile-based action for the PSU dataset.

The PSU dataset [76] contains 328 video clips of human profiles with four basic actions recorded in two views using RGBD cameras (Kinect ver.1). The videos were simultaneously captured and synchronized between views. The profile-based actions consisted of standing/walking, sitting, stooping, and lying. Two scenarios, one in a work room for training and another in a living room for testing, were performed.  Figure 4 shows an example of each scenario, together with the viewpoints covered.

**Figure 5:**
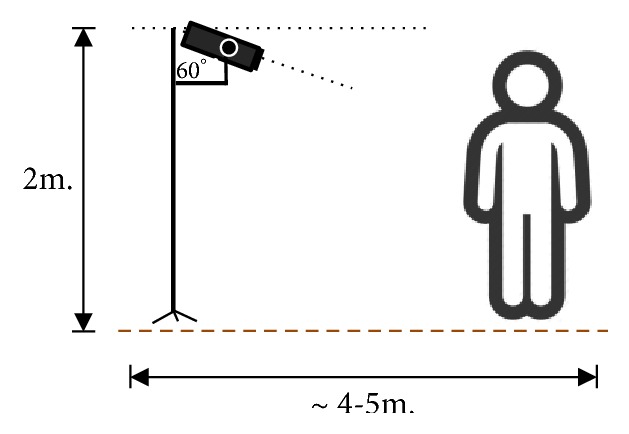
General camera installation and setup.

Two Kinect cameras were set overhead at 60° to the vertical line, each at the end of a 2 m pole. RGB and depth information from multiviews was taken from the stationary cameras with varying viewpoints to observe the areas of interest. The operational range was about 3-5.5 m from the cameras to accommodate a full body image, as illustrated in Figure 5. The RGB resolution for the video dataset was 640×480, while depths at 8 and 24 bits were also of the same resolution. Each sequence was performed by 3-5 actors, having no less than 40 frames of background at the beginning to allow motion detection using any chosen background subtraction technique. The frame rate was about 8-12 fps.

**Figure 6:**
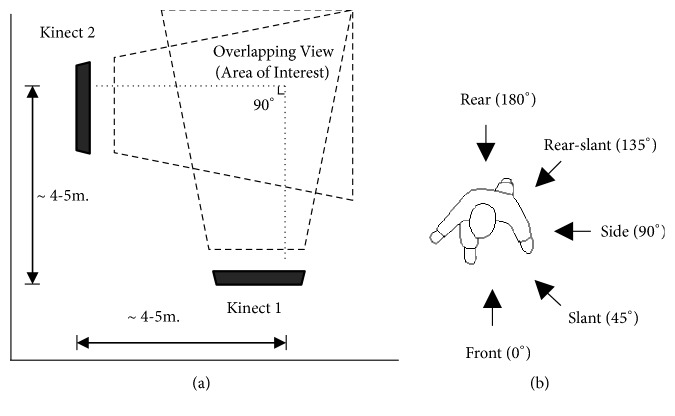
Scenario in the work room. (a) Installation and scenario setup from top view. (b) Orientation angle to object.

*(i) Scenario in the Work Room (Training Set)*. As illustrated in Figure 6(a), the two cameras' views are perpendicular to each other. There are five angles of object orientation: front (0°), slant (45°), side (90°), rear-slant (135°), and rear (180°), as shown in Figure 6(b). A total of 8,700 frames were obtained in this scenario for training.

**Figure 7:**
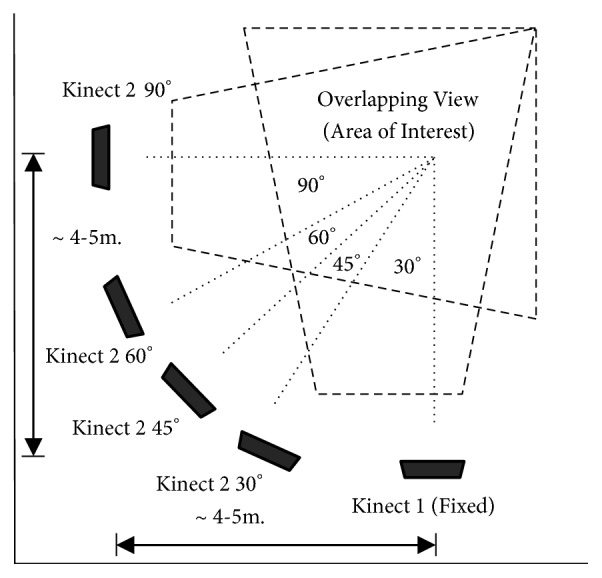
Installation and scenario of the living room setup (four positions of Kinect camera 2, at 30°, 45°, 60°, and 90°, and stationary Kinect camera 1).

*(ii) Scenario in the Living Room (Testing Set)*. This scenario, illustrated in Figure 7, involves one moving Kinect camera at four angles: 30°, 45°, 60°, and 90°, while another Kinect camera remains stationary. Actions are performed freely in various directions and positions within the area of interest. A total of 10,720 frames of actions were tested.

#### 3.1.1. Evaluation of the Number of Layers

**Figure 8:**
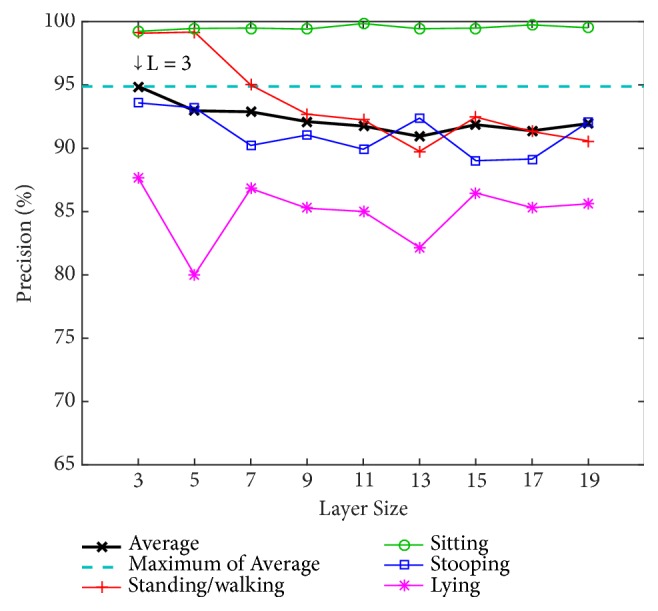
Precision by layer size for the four postures using an artificial neural network (ANN) on the PSU dataset.

**Figure 9:**
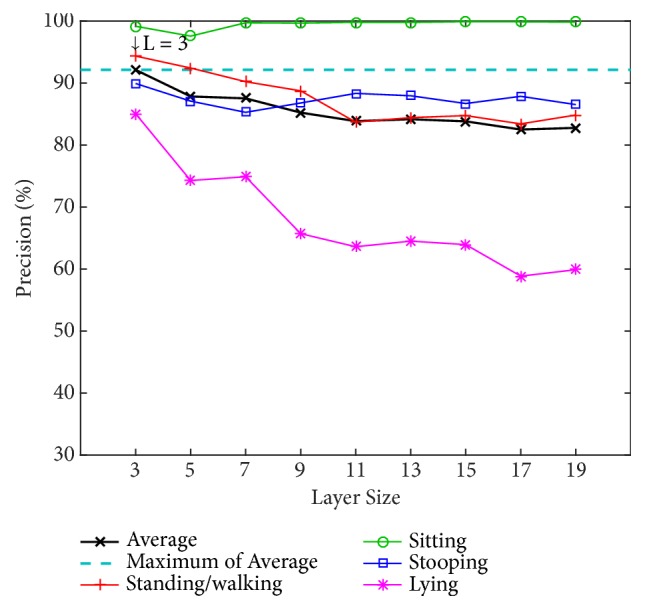
Precision by layer size for the four postures using support vector machine (SVM) on the PSU dataset.

We determine the appropriate number of layers by testing our model with different numbers of layers (L) using the PSU dataset. The numbers of layers for testing are 3, 5, 7, 9, 11, 13, 15, 17, and 19. The alpha value (*α*) is instinctively fixed at 0.7 to find the optimal value of the number of layers. Two classification methods are used for training and testing: an artificial neural network (ANN) with a back-propagation algorithm over 20 nodes of hidden layers and a support vector machine (SVM) using a radial basis function kernel along with C-SVC. The results using ANN and SVM are shown in Figures 8 and 9, respectively.

Figure 8 shows that the 3-layer size achieves the highest average precision of 94.88% using the ANN and achieves 92.11% using the SVM in Figure 9. Because the ANN performs better, in the tests that follow, the evaluation of our model will be based on this layer size together with the use of the ANN classifier.

#### 3.1.2. Evaluation of Adjustable Parameter *α*

For the weighted mass of dimension feature, ($\overset{-}{\omega }[k]$), the adjustment parameter alpha (*α*)—the value for the weight between the inverse depth density of layers (Q[*k*]) and the density of layer (*ρ*[*k*])—is employed. The optimal *α* value is determined to show the improved feature that uses inverse depth to reveal hidden volume in some parts and the normal volume. The experiment is carried out by varying alpha from 0 to 1 at 0.1 intervals.

**Figure 10:**
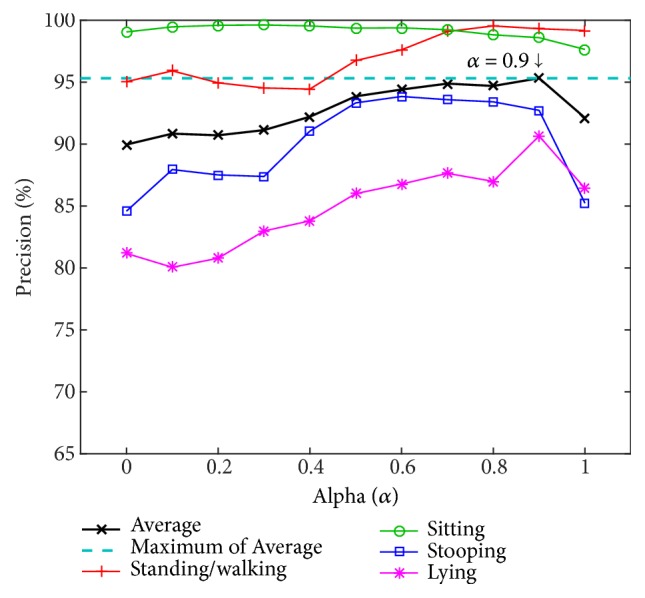
Precision versus *α* (the adjustment parameter for weighting between Q[*k*] and *ρ*[*k*] when L = 3, from the PSU dataset).

Figure 10 shows the precision of action recognition using a 3-layer size and the ANN classifier versus the alpha values. In general, except for the sitting action, one may note that as the portion of inverse depth density of layers (Q[*k*]) is augmented, precision increases. The highest average precision is 95.32% at *α* = 0.9, meaning that 90% of the inverse depth density of layers (Q[*k*]) and 10% of the density of layers (*ρ*[*k*]) are an optimal proportion. When *α* is above 0.9, all precision values drop. The trend for sitting action is remarkably different from others in that precision always hovers near the maximum and gradually but slightly decreases when *α* increases.

**Figure 11:**
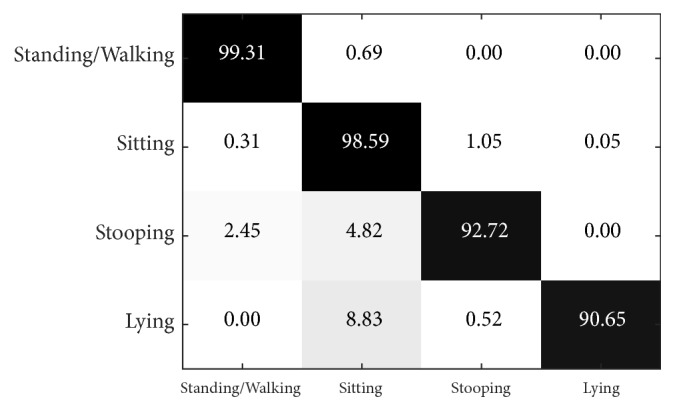
Confusion matrix for multiview recognition in the PSU dataset when L = 3 and *α* = 0.9.

Figure 11 illustrates the multiview confusion matrix of action precisions when L = 3 and *α* = 0.9, using the PSU dataset. We found that standing/walking action had the highest precision (99.31%), while lying only reached 90.65%. The classification error of the lying action depends mostly on its characteristic that the principal axis of the body is aligned horizontally, which works against the feature model. In general, the missed classifications of standing/walking, stooping, and lying actions were mostly confused with the sitting action, accounting for 0.69%, 4.82%, and 8.83% of classifications, respectively. Nevertheless, the precision of sitting is relatively high at 98.59%.

#### 3.1.3. Comparison of Single View/Multiview

**Figure 12:**
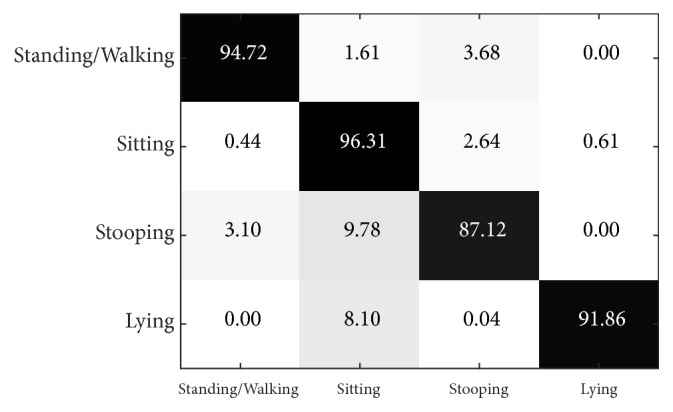
Confusion matrix of single-view Kinect 1 recognition (stationary camera) for the PSU dataset when L = 3 and *α* = 0.9.

**Figure 13:**
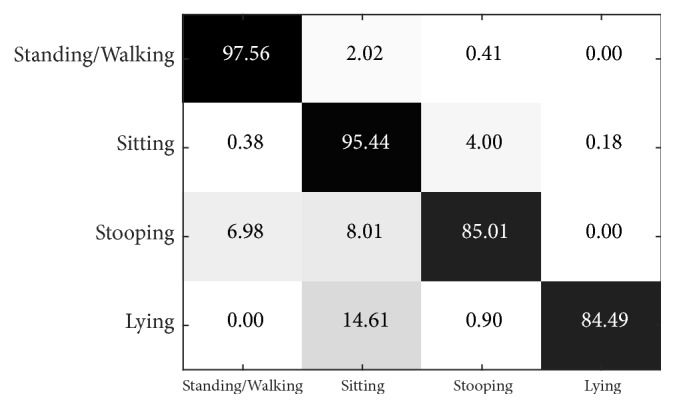
Confusion matrix of single-view Kinect 2 recognition (moving camera) for the PSU dataset when L = 3 and *α* = 0.9.

For the sake of comparison, we also evaluate tests in single-view recognition for the PSU dataset for L = 3 and *α* = 0.9 in the living room scene, similar to that used to train the classification model for the work room.  Figure 12 shows the results from the single-view Kinect 1 (stationary camera), while Figure 13 shows those from the single-view Kinect 2 (moving camera).

Results show that the single-view Kinect 1, which is stationary, performs slightly better than the single-view Kinect 2, which is moving (average precision of 92.50% compared to 90.63%). The stationary camera gives the best results for sitting action, while for the moving camera, the result is best for the standing/walking action. It is worth noting that the stationary camera yields a remarkably better result for lying action than the moving one.

**Figure 14:**
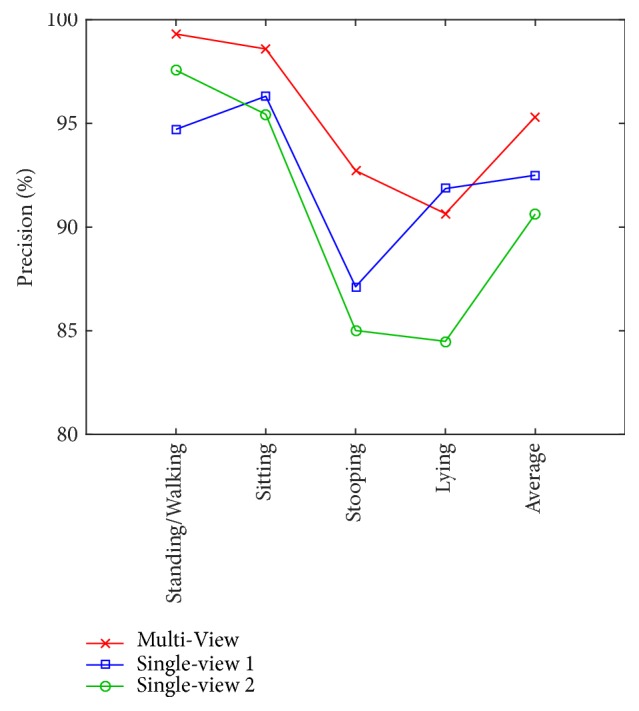
Comparison of precision from multiview, single-view 1, and single-view 2 for the PSU dataset when L = 3 and *α* = 0.9.

Figure 14 shows the precision of each of the four postures, together with that of the average, for the multiview and the two single views. On average, the result is best accomplished with the use of the multiview and is better for all postures other than the lying action. In this regard, single-view 1 yields a slightly better result, most probably due to its stationary viewpoint toward the sofa, which is perpendicular to its line of sight.

#### 3.1.4. Comparison of Angle between Cameras

**Figure 15:**
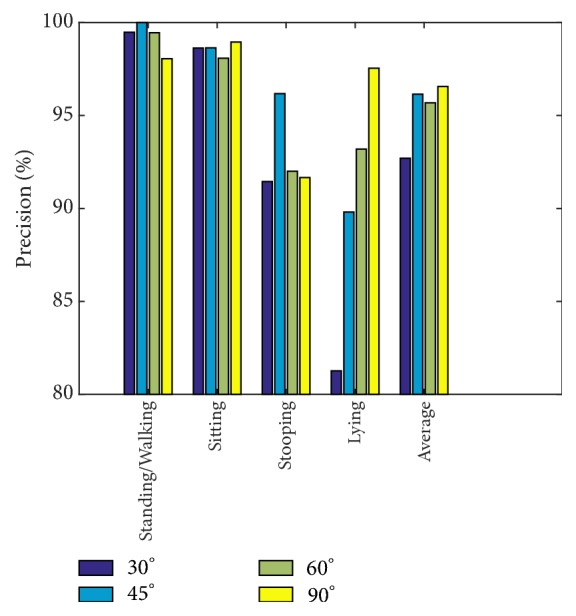
Precision comparison graph on different angles in each action.

As depicted earlier in Figure 7, the single-view Kinect 1 camera is stationary, while the single-view Kinect 2 camera is movable, adjusted to capture viewpoints at 30°, 45°, 60°, and 90° to the stationary camera. We test our model on these angles to assess the robustness of the model on the four postures. Results are shown in Figure 15.

In Figure 15, the lowest average precision result occurs at 30°—the smallest angle configuration between the two cameras. This is most probably because the angle is narrow and thus not much additional information is gathered. For all other angles, the results are closely clustered. In general, standing/walking and sitting results are quite consistent at all angles, while lying and stooping are more affected by the change.

#### 3.1.5. Evaluation with NW-UCLA Trained Model

**Figure 16:**
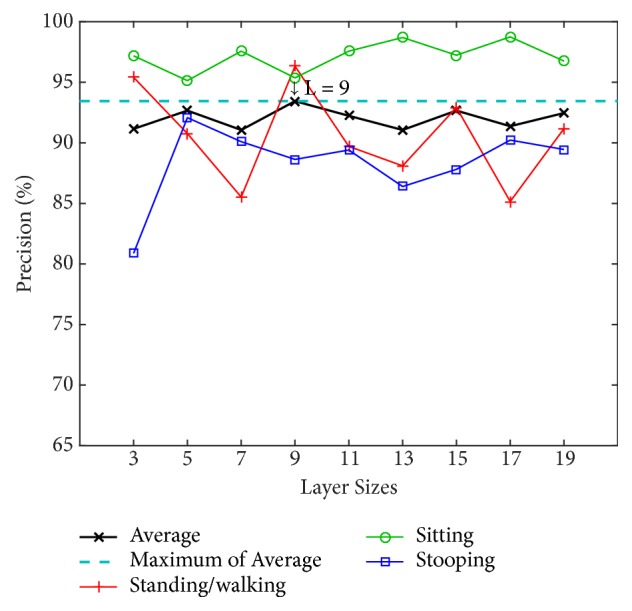
Precision by layer size for the PSU dataset when using the NW-UCLA-trained model.

In addition, we tested the PSU dataset in living room scenes using the model trained on the NW-UCLA dataset [63].  Figure 16 illustrates that the 9-layer size achieves the highest average precision at 93.44%. Sitting gives the best results and the highest precision, up to 98.74% when L = 17. However, sitting at low layers also gives good results, for example, L = 3 at 97.18%, while the highest precision for standing/walking is 95.40%, and the lowest precision is 85.16%. The lowest precision for stooping is 92.08% when L = 5.

**Figure 17:**
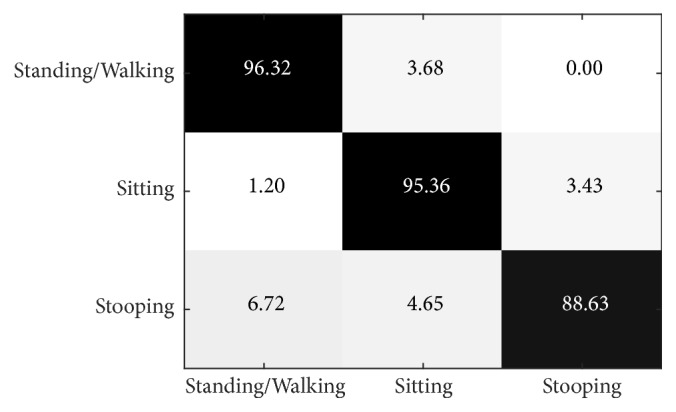
Confusion matrix of 2 views for the PSU dataset (using the NW-UCLA-trained model) when L = 9.

Figure 17 shows the results when L = 9, which illustrates that standing/walking gives the highest precision at 96.32%, while bending gives the lowest precision at 88.63%.

**Figure 18:**
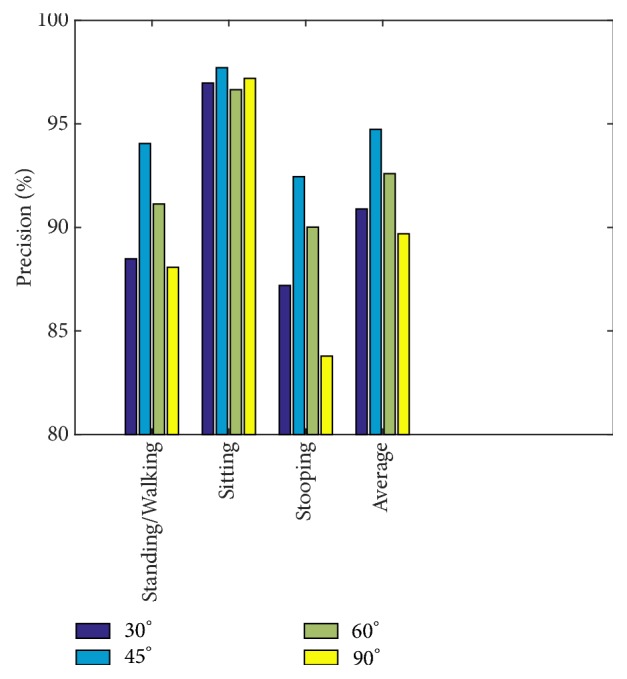
Precision comparison graph of different angles in each action when using the NW-UCLA-trained model.

In addition, we also compare precision of different angles between cameras, as shown in Figure 18. The result shows that the highest precision on average is 94.74% at 45°, and the lowest is 89.69% at 90°.

In general, the precision of all actions is highest at 45° and decreases as the angle becomes larger. However, the results obtained using the PSU-trained model show a different trend, where a larger angle provides better results.

#### 3.1.6. Evaluation of Time Consumption

Time consumption evaluation, excluding interface and video showing time, is conducted using the OpenMP wall clock. Our system is tested on a normal PC (Intel® Core™ i5 4590 at 3.30 GHz with 8 GB DDR3). We use the OpenCV library for computer vision, the OpenMP library for parallel processing, and CLNUI to capture images from the RGBD cameras. The number of layers and the classifier are tested using 10,720 action frames from the living room scene.

On average, the time consumption is found to be approximately 15 ms per frame or a frame rate of around 63 fps. As detailed in Table 3, the number of layers and the type of classifier affect the performance only slightly. In addition, we compare serial processing with parallel processing, which divides a single process into threads. The latter is found to be 1.5507 times faster than the former. It was noted that thread initialization and synchronization consume a portion of the computation time.

### 3.2. Experiment on the NW-UCLA Dataset

The NW-UCLA dataset [63] is used to benchmark our method. This dataset is similar to our work for the multiview action 3D PSU dataset taken at different viewpoints to capture the RGB and depth images. The NW-UCLA dataset covers nearly ten actions including* stand up*,* walk around*,* sit down*, and* bend to pick up item*, but it lacks a* lie down* action. Actions in this dataset are marked in colors. To test our method, the motion detection step for segmenting movement is replaced by a specific color segmentation to obtain the human structure. The rest of the procedure stays the same.

Only actions of interest are selected for the test—its transition actions are excluded. For example, standing/walking frames are extracted from stand up and walk around; sitting frames are selected from sit down and stand up; and stooping is extracted from* pick up with one hand* and* pick up with both hands*.

**Figure 19:**
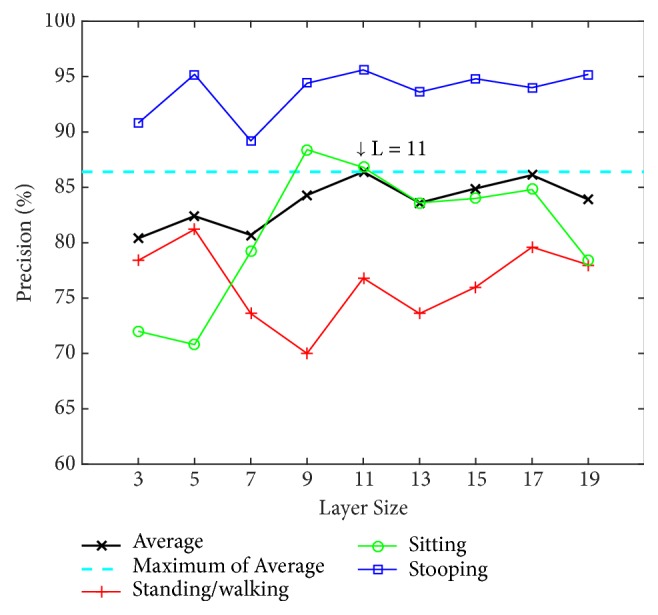
Precision of action by layer size on NW-UCLA dataset.

Our method employs the model learned using the PSU dataset in the work room scenario to test the NW-UCLA dataset. All parameters in the test are the same except that alpha is set to zero due to variations in depth values. The experiment is performed for various numbers of layers from L = 3 to L = 19. Test results on the NW-UCLA dataset are shown in Figure 19.

**Figure 20:**
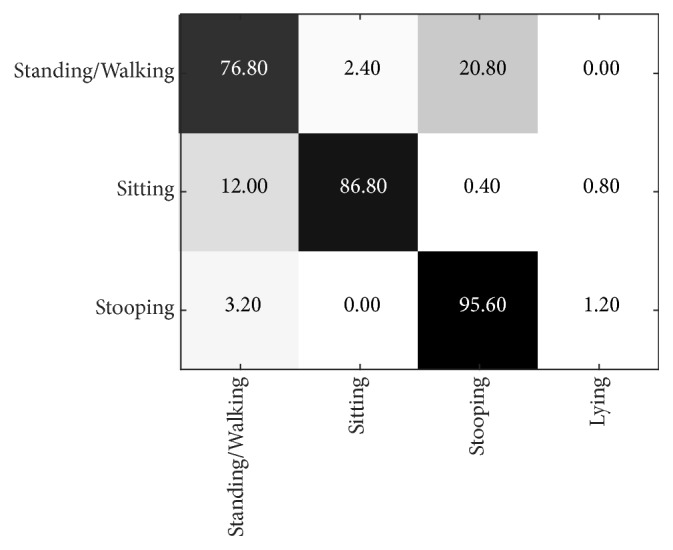
Confusion matrix of test results on the NW-UCLA 3D dataset when L = 11.

From Figure 19, the maximum average precision (86.40%) of the NW-UCLA dataset is obtained at layer L =11, in contrast to the PSU dataset at L = 3. Performance for stooping is generally better than other actions and peaks at 95.60%. As detailed in Figure 20, standing/walking gives the lowest precision at 76.8%. The principal cause of low precision is that the angle of the camera and its captured range are very different from the PSU dataset. Compared with the method proposed by NW-UCLA, our method performs better by up to 13 percentage points, from an average of 73.40% to 86.40%, as shown in Table 4. However, to be fair, many more activities are considered by the NW-UCLA than ours which focuses only on four basic actions and hence its disadvantage by comparison.

### 3.3. Experiment on the i3DPost Dataset

The i3DPost dataset [77] is an RGB multiview dataset that contains 13 activities. The dataset was captured by 8 cameras from different viewpoints with 45° between cameras, performed by eight persons for two sets at different positions. The background images allow the same segmentation procedure to build the* nondepth feature vector* for recognizing profile-based action.

The testing sets extract only target actions from temporal activities, including standing/walking, sitting, and stooping from* sit-standup*,* walk*,* walk-sit*, and* bend*.

#### 3.3.1. Evaluation of i3DPost Dataset with PSU-Trained Model

Firstly, i3DPost is tested by the PSU-trained model from 2 views at 90° between cameras on different layer sizes.

**Figure 21:**
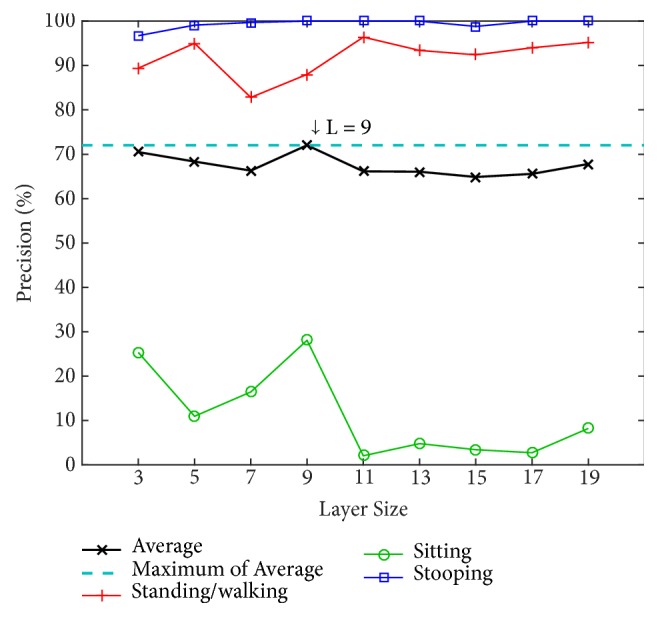
Precision of i3DPost dataset by layer size when using the PSU-trained model.

Figure 21 shows the testing results of the i3DPost dataset using the PSU-trained model. The failed prediction for sitting shows a precision of only 28.08% at L = 9. By observation, the mistake is generally caused by the action of sitting that looks like a squat in the air, which is predicted as standing. In the PSU dataset, sitting is done on a bench/chair. On the other hand, standing/walking and stooping are performed with good results of about 96.40% and 100% at L = 11, respectively.

**Figure 22:**
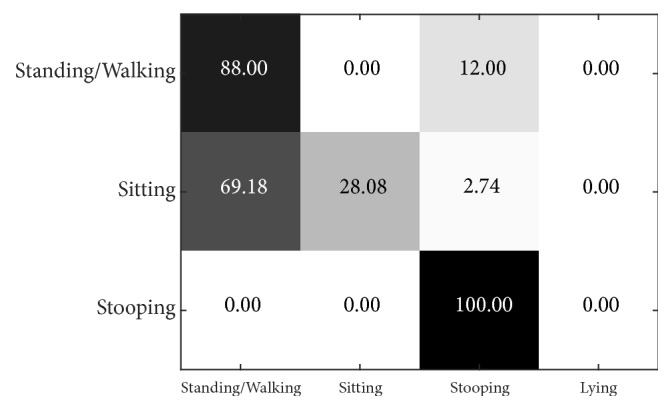
Confusion matrix of 2 views for i3DPost dataset using PSU-trained model when L = 9.

Figure 22 shows the multiview confusion matrix when L = 9. Sitting is most often confused with standing/walking (69.18% of cases), and standing/walking is confused with stooping (12.00% of cases).

#### 3.3.2. Training and Evaluation Using i3DPost

From the last section, i3DPost evaluation using the PSU-trained model resulted in missed classification for the sitting action. Accordingly, we experimented with our model by training and evaluating using only the i3DPost; the first dataset is used for testing and the second for training. The initial testing is performed in 2 views.

**Figure 23:**
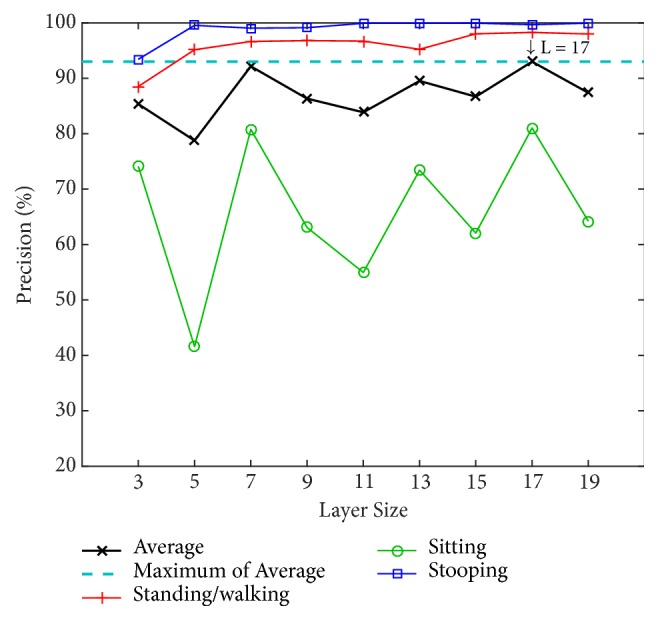
Precision of i3DPost dataset with new trained model by layer size.

**Figure 24:**
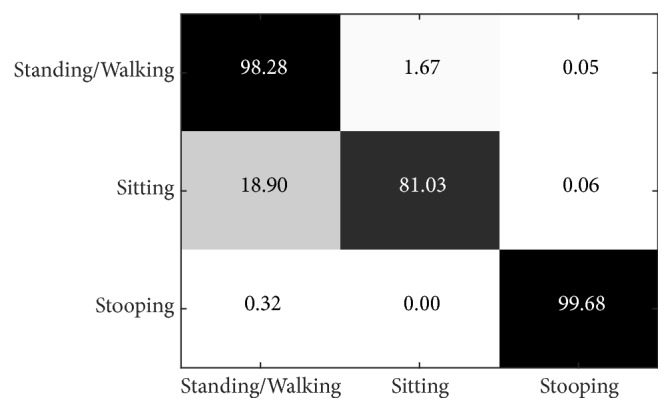
Confusion matrix of test result on the 2-view i3DPost dataset when L = 17.

Figures 23 and 24 show the results of each layer size from 2 views. The 17 layers achieve the highest precision at 93.00% on average (98.28%, 81.03%, and 99.68% for standing/walking, sitting, and stooping, resp.). In general, standing/walking and stooping achieve good precision of above 90%, except at L = 3. However, the best precision for sitting is only 81.03%, where most wrong classifications are defined as standing/walking (18.90% of cases), and the lowest precision is at 41.59% when L = 5. We noticed that the squat still affects performance.

**Figure 25:**
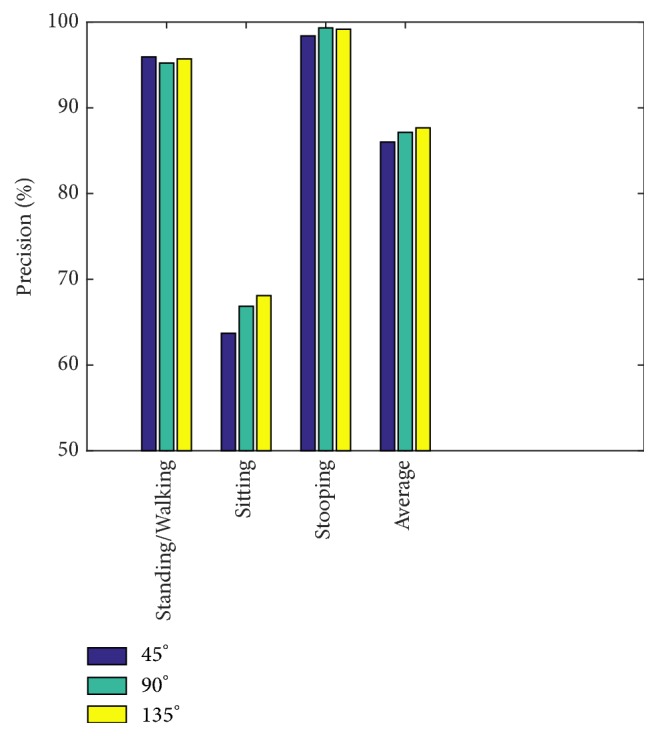
Precision comparison graph for different angles in the i3DPost.

In 2-view testing, we couple the views for different angles between cameras, such as 45°, 90°, and 135°.  Figure 25 shows that, at 135°, the performance on average is highest, and the lowest performance is at 45°. In general, a smaller angle gives lower precision; however, for sitting, a narrow angle may reduce precision dramatically.

**Figure 26:**
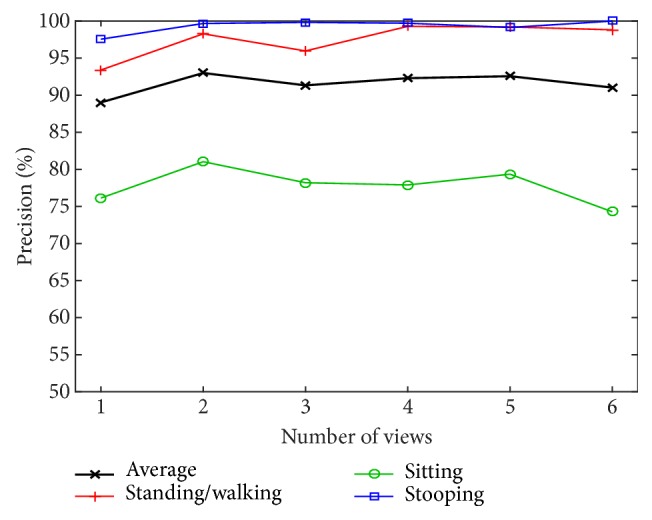
Precision in i3DPost dataset with new trained model by the number of views.

In addition, we perform the multiview experiments for various numbers of views from one to six in order to evaluate our multiview model, as shown in Figure 26.

Figure 26 shows the precision according to the number of views. The graph reports the maximum precision versus the different number of views from one to six views, which are 89.03%, 93.00%, 91.33%, 92.30%, 92.56%, and 91.03% at L = 7, L = 17, L = 17, L = 7, and L = 13, respectively. We noticed that the highest precision is for 2 views. In general, the performance increases when the number of views increases, except for sitting. Moreover, the number of layers that give maximum precision is reduced as the number of views increases. In conclusion, only two or three views from different angles are necessary for obtaining the best performance.

We compared our method with a similar approach [59], based on a posture prototype map and results voting function with a Bayesian framework for multiview fusion.  Table 5 shows the comparison results. The highest precisions of our method and the comparison approach are 99.68% and 100% for stooping and bend, respectively. Likewise, the lowest precisions are 81.03% and 87.00% for the same actions. However, for walking/standing, our approach obtains better results. On average, the comparison approach performs slightly better than our method.

## 4. Comparison with Other Studies

Our study has now been presented with other visual profile-based action recognition studies and also compared with other general action recognition studies.

### 4.1. Precision Results of Different Studies

Table 6 shows precision results of various methods emphasizing profile-based action recognition. Note that the methods employed different algorithms and datasets; thus results are presented only for the sake of studying their trends with respect to actions. Our method is tested on the PSU dataset. Our precision is highest on walking and sitting actions, quite good on the standing action, quite acceptable on the lying action, but poor on the stooping action. It is not possible to compare on precision due to lack of information on the performance of other methods. However, we can note that each method performs better than others on different actions; for example, [74] is good for standing, and [73] is a better fit for sitting.

### 4.2. Comparison with Other Action Recognition Studies

Table 7 compares the advantages and disadvantages of some previous studies concerning action recognition acquired according to the criteria of their specific applications. In the inertial sensor-based approach, sensors/devices are attached to the body and hence monitoring is available everywhere, for the inconvenience of carrying them around. This approach gives high privacy but is highly complex. In an RGB vision-based approach, sensors are not attached to the body, and hence it is less cumbersome. Though not so complex, the main drawback of this method is the lack of privacy. In this regard, depth-based views may provide more privacy. Although rather similar in comparison in the table, the multiview approach can cope with some limitations, such as vision coverage and continuity or obstruction, which are common in the single-view approach, as described in some detail in Introduction.

- As for our proposed work, it can be seen from the table that the approach is in general quite good in comparison; in addition to being simple, it offers a high degree of privacy and other characteristics such as flexibility, scalability, and robustness are at similar levels, if not better, and no calibration is needed, which is similar to most of the other approaches.
- However, the two cameras for our multiview approach still have to be installed following certain specifications, such as the fact that the angle between cameras should be more than 30°.

## 5. Conclusion/Summary and Further Work

In this paper, we explore both the inertial and visual approaches to camera surveillance in a confined space such as a healthcare home. The less cumbersome vision-based approach is studied in further detail for single-view and multiview RGB depth-based and for non-RGB depth-based approaches. We decided on a multiview, non-RGB depth-based approach for privacy and have proposed a layer fusion model, which is representation-based model that allows fusion of features and information by segmenting parts of the object into vertical layers.

We trained and tested our model on the PSU dataset with four postures (standing/walking, sitting, stooping, and lying) and have evaluated the outcomes using the NW-UCLA and i3DPost datasets on available postures that could be extracted. Results show that our model achieved an average precision of 95.32% on the PSU dataset—on a par with many other achievements, if not better, 93.00% on the i3DPost dataset, and 86.40% on the NW-UCLA dataset. In addition to flexibility of installation, scalability, and noncalibration of features, one advantage over most other approaches is that our approach is simple, while it contributes good recognition on various viewpoints with a high speed of 63 frames per second, suitable for application in a real-world setting.

In further research, a spatial-temporal feature is interesting and should be investigated for more complex action recognition, such as waving and kicking. Moreover, reconstruction of 3D structural and bag-of-visual-word models is also of interest.

## Figures and Tables

**Table 1:** Sampled human profile depth in various views and actions.

Action/view	***I***_***mo***_	*ρ* [*k*]	D [*k*]	D_*i*_[*k*]	Action/view	***I***_***mo***_	*ρ* [*k*]	D [*k*]	D_*i*_[*k*]
Standing-walking/front	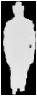	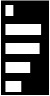	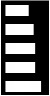	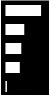	Stooping/front	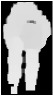	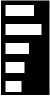	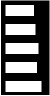	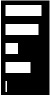
Standing-walking/slant	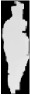	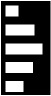	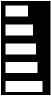	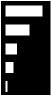	Stooping/slant	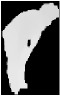	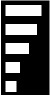	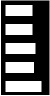	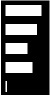
Standing-walking/side	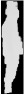	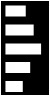	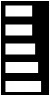	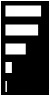	Stooping/side	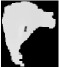	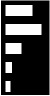	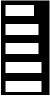	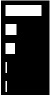
Standing-walking/back slant	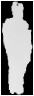	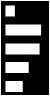	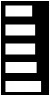	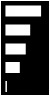	Stooping/back slant	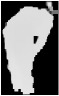	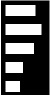	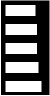	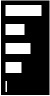
Standing-walking/back	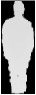	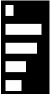	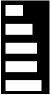	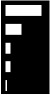	Stooping/back	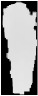	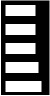	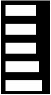	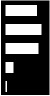
Sitting/front	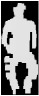	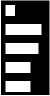	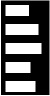	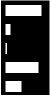	Lying/front	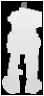	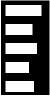	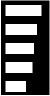	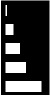
Sitting/slant	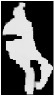	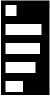	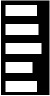	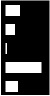	Lying/slant	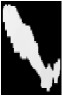	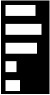	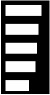	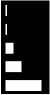
Sitting/side	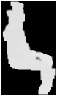	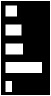	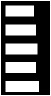	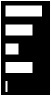	Lying/side	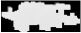	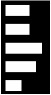	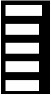	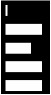
Sitting/back slant	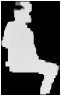	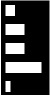	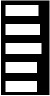	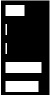	Lying/back slant	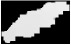	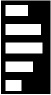	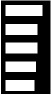	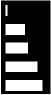
Sitting/back	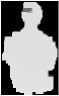	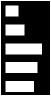	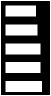	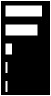	Lying/back	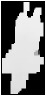	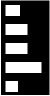	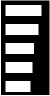	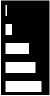

Note: (I) *ρ*[*k*] is density of layer. (II) D  [*k*] is real-range depth of layer. (III) D_*i*_[*k*] is inverse depth of layer.

**Table 2:** Sampled features in single view and fused features in multiview.

Action/view	Camera 1		Camera 2		$\overset{-}{\omega }[k]$
	***I***_***mo***_	*Ζ*[*k*] | *α* = 0.9	***I***_***mo***_	*Ζ*[*k*] | *α* = 0.9	
(I) Standing-walking/front	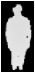	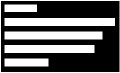	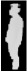	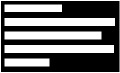	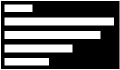
(II) Standing-walking/slant	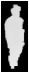	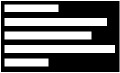	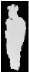	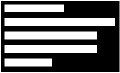	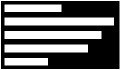
(III) Standing-walking/side	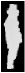	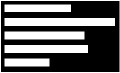	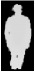	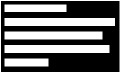	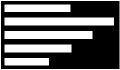
(IV) Standing-walking/back slant	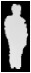	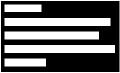	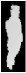	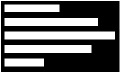	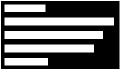
(V) Standing-walking/back	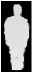	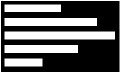	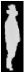	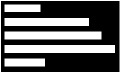	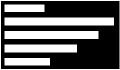
(VI) Sitting/front	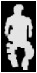	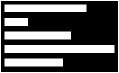	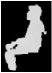	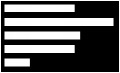	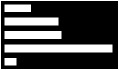
(VII) Sitting/slant	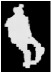	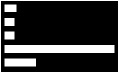	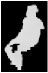	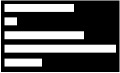	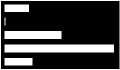
(VIII) Sitting/side	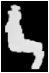	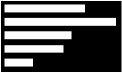	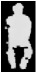	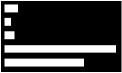	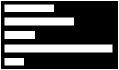
(IX) Sitting/back slant	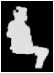	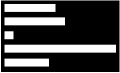	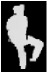	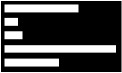	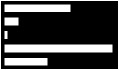
(X) Sitting/back	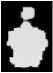	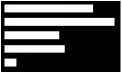	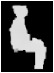	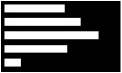	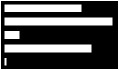
(XI) Stooping/front	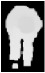	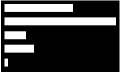	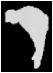	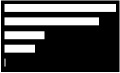	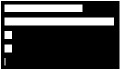
(XII) Stooping/slant	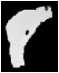	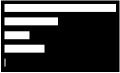	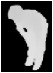	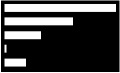	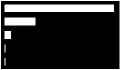
(XIII) Stooping/side	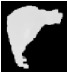	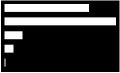	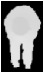	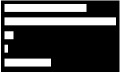	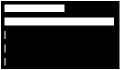
(XIV) Stooping/back slant	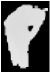	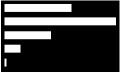	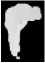	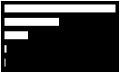	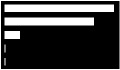
(XV) Stooping/back	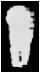	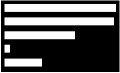	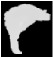	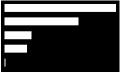	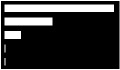
(XVI) Lying/front	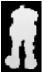	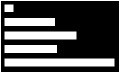	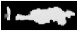	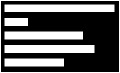	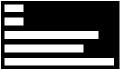
(XVII) Lying/slant	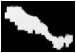	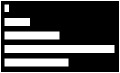	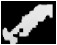	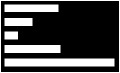	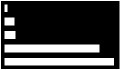
(XVIII) Lying/side	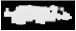	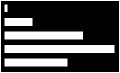	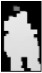	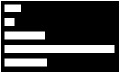	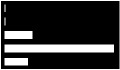
(XIX) Lying/back slant	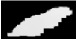	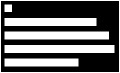	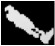	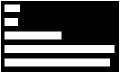	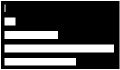
(XX) Lying/back	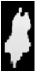	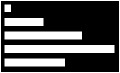	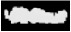	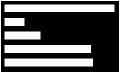	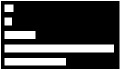

Note: (I) Z[*k*] is weighted depth density of layers which uses *α* for adjustment between Q[*k*] and *ρ*[*k*]. (II) $\overset{-}{\omega }[k]$, fused features from multiview, called the weighted mass of dimension feature.

**Table 3:** Time consumption testing on different numbers of layers and different classifiers.

Classifier	Time consumption (ms)/frame									Frame rate (fps)		
	L=3			L=11			L=19			L=3	L=11	L=19
	Min	Max	Avg	Min	Max	Avg	Min	Max	Avg	Avg	Avg	Avg
ANN	13.95	17.77	15.08	14.12	20.58	15.18	14.43	20.02	15.85	66.31	65.88	63.09
SVM	14.01	17.40	15.20	14.32	18.97	15.46	14.34	19.64	15.71	65.79	64.68	63.65

Note: L stands for the number of layers. ANN, artificial neural network; SVM, support vector machine.

**Table 4:** Comparison between NW-UCLA and our recognition systems on the NW-UCLA dataset.

NW-UCLA recognition action [63]	Precision (%)	Our recognition action	Precision (%)
Walk around	77.60	Walking/standing	76.80
Sit down	68.10	Sitting	86.80
Bend to pick item (1 hand)	74.50	Stooping	95.60
Average	73.40	Average	86.40

**Table 5:** Comparison between our recognition systems and [59] on the i3DPost dataset.

Recognition of similar approach [59]	Precision (%)	Our recognition action	Precision (%)
Walk	95.00	Walking/standing	98.28
Sit	87.00	Sitting	81.03
Bend	100.0	Stooping	99.68
Average	94.00	Average	93.00

**Table 6:** Precision results of our study and others emphasizing profile-based action recognition using different methods and datasets.

Method	Precision rate of action (%)					
	*Standing*	*Walking*	*Sitting*	*Stooping*	*Lying*	*Average*
P. Chawalitsittikul et al. [70]	98.00	98.00	93.00	94.10	98.00	96.22
N. Noorit et al. [71]	99.41	80.65	89.26	94.35	100.0	92.73
M. Ahmad et al. [72]	-	89.00	85.00	100.0	91.00	91.25
C. H. Chuang et al. [73]	-	92.40	97.60	95.40	-	95.80
G. I. Parisi et al. [74]	96.67	90.00	83.33	-	86.67	89.17
N. Sawant et al. [75]	91.85	96.14	85.03	-	-	91.01
Our method on PSU dataset	99.31	99.31	98.59	92.72	90.65	95.32

**Table 7:** Comparison of some action recognition approaches based on ease of use, perspective robustness, and real-world application conditions.

Approach	Flexible calibration	View scalability	Perspective robustness	Real-world Application Condition		
				Complexity	Cumbersomeness from sensor/device attachment	Privacy
Inertial sensor-based [1–12]	N/A	N/A	High	Highly complex	Cumbersome	High
RGB vision-based [13, 17, 21–47, 71–73, 75]	Low-moderate	Low-moderate	Low-moderate	Simple-moderate	Not cumbersome	Lacking
Depth-based Single-view [14–16, 18–20, 70, 74]	Low-moderate	Low-moderate	Low-moderate	Depends	Not cumbersome	Moderate-high
RGB/depth-based multiview [48–68]	Low-moderate	Low-moderate	Low-moderate	Depends	Not cumbersome	Depends
**Our work, depth-based multiview **	**Moderate-high**	**Moderate-high**	**High**	**Simple**	**Not cumbersome**	**High**

## Data Availability

The PSU multiview profile-based action dataset is available at [74], which is authorized only for noncommercial or educational purposes. The additional datasets to support this study are cited at relevant places within the text as references [63] and [75].
